# Salvage CD20-SD-CART therapy in aggressive B-cell lymphoma after CD19 CART treatment failure

**DOI:** 10.3389/fonc.2024.1376490

**Published:** 2024-06-25

**Authors:** Fei Xue, Peihao Zheng, Fan Yang, Rui Liu, Shaomei Feng, Yuelu Guo, Hui Shi, Lixia Ma, Biping Deng, Teng Xu, Jiecheng Zhang, Qi Zhou, Xiaoyan Ke, Kai Hu

**Affiliations:** ^1^ Department of Lymphoma and Myeloma Research Center, Beijing Gobroad Boren Hospital, Beijing, China; ^2^ Cytology Laboratory, Beijing Gobroad Boren Hospital, Beijing, China; ^3^ Department of Hospital Management, Gobroad Medical Group, Beijing, China; ^4^ Clinical Research Centre, GoBroad Healthcare Group, Beijing, China

**Keywords:** B-cell lymphoma, CD19, CD20CART, CART treatment failure, salvage therapy

## Abstract

**Background and aims:**

Patients with relapsed/refractory aggressive B-cell lymphoma(r/r aBCL)who progressed after CD19-specific chimeric antigen receptor T-cell therapy (CD19CART) had a poor prognosis. Application of CAR T-cells targeting a second different antigen (CD20) expressed on the surface of B-cell lymphoma as subsequent anti-cancer salvage therapy (CD20-SD-CART) is also an option. This study aimed to evaluate the survival outcome of CD20-SD-CART as a salvage therapy for CD19 CART treatment failure.

**Methods:**

This retrospective cohort study enrolled patients with aBCL after the failure of CD19 CART treatment at Beijing Gobroad Boren Hospital from December 2019 to May 2022. Patients were subsequently treated with CD20CART therapy or non-CART therapy (polatuzumab or non-polatuzumab).

**Results:**

A total of 93 patients were included in the study, with 54 patients receiving CD20-SD-CART therapy. After a median follow-up of 18.54 months, the CD20-SD-CART group demonstrated significantly longer median progression-free survival (4.04 months vs. 2.27 months, p=0.0032) and median overall survival (8.15 months vs. 3.02 months, p<0.0001) compared to the non-CART group. The complete response rate in the CD20-SD-CART group (15/54, 27.8%) was also significantly higher than the non-CART group (3/38, 7.9%, p=0.03). Multivariate analysis further confirmed that CD20CART treatment was independently associated with improved overall survival (HR, 0.28; 95% CI, 0.16–0.51; p<0.0001) and progression-free survival (HR, 0.46; 95% CI, 0.27–0.8; p=0.005).

**Conclusion:**

CD20-SD-CART could serve as an effective therapeutic option for patients with relapsed or refractory aggressive B-cell lymphoma after CD19CART treatment failure.

## Introduction

CD19-specific chimeric antigen receptor T-cell therapy (CD19 CART) has demonstrated significant improvements in overall response rates (ORRs) and complete response rates (CRRs) for patients with relapsed/refractory aggressive B-cell lymphoma (r/r aBCL), with reported rates ranging from 52.0% to 83% (1–4). However, despite these promising results, approximately 60% of patients experience disease progression within one year following CD19 CART treatment (5–9). Furthermore, the overall survival for patients who progressed after CD19 CART therapy is notably poor, with a median overall survival of approximately 5.2 to 6 months (6–8). There is an urgent need to develop effective salvage therapies for r/r aBCL patients.

Following the failure of CD19 CART therapy, various non-CART treatment options, such as chemotherapy, radiotherapy, targeted drugs (polatuzumab-vedotin-based, immune checkpoint inhibitors, and bispecific antibodies), have been employed for further anti-cancer therapies (5–7, 10–12). However, the efficacy of these treatment modalities varies, and there is currently limited available data and no consensus on the optimal therapy for this population.

In addition to CD19, CD20 is another B-cell antigen expressed on the surface of most B-cell malignancies. CD20-targeted CART therapy has shown effectiveness in treating r/r aBCL patients after chemotherapy, suggesting a potential salvage treatment strategy for those who have poor response to CD19 CART therapy (13, 14). However, the efficacy of CD20-targeted CART therapy in the context of CD19 CART failure remains unknown.

This study aimed to investigate the survival outcome of CD20-targeted CART therapy as a salvage treatment for aBCL patients after failure of CD19 CART treatment.

## Methods

### Study design and patients

This study retrospectively analyzed aBCL patients who had received CD19 CART therapy (clinical Trials#: ChiCTR2100055062) and then progressed (relapsed/refractory to CD19 CART) at Beijing Gobroad Boren Hospital from December 2019 to May 2022. This study was approved by the Institutional Review Board of Boren Hospital (approval number: 20191225-PJ-003) and was conducted according to the principles of the Declaration of Helsinki. Written informed consent was obtained from each patient.

The inclusion criteria of this study were: 1) ≥18 years old; 2) patients with diffuse large B-cell lymphoma, not otherwise specified (DLBCL, NOS), Burkitt lymphoma (BL), transformed follicular lymphoma (tFL), high-grade B-cell lymphomas (HGBL) (15) after two or more lines of systemic therapy received murinized second-generation anti-CD19 CAR-T cells with signals provided by costimulatory molecules 4–1BB and CD3-zeta, and 3) patients who did not achieve complete remission (CR) or partial remission (PR) after CD19 CART treatment (refractory to CD19 CART), or experienced disease progression after initially achieving CR/PR (relapsed to CD19 CART). The exclusion criteria were 1) patients showing sustained remission following CD19CART treatment, 2) patients who received CD19 CART and other drugs simultaneously, and 3) patients who experienced progression after CD19 CART without subsequently receiving anti-cancer therapy.

The selection of therapy is determined by the positive expression status of tumor tissue antigens (CD20 positive, ≥90%), physical condition (ECOG <4 or ECOG=4 but demanding further anti-tumor treatment), and wiliness of included patients. Patients were divided into using CAR T-cells targeting a second different antigen (CD20) (CD20-SD-CART) group and non-CART group, depending on the subsequent anti-cancer treatment.

### Treatment

CD20CART cells were manufactured using cryopreserved autologous peripheral blood mononuclear cells stored when patients enrolled/consented to CD19 CART therapy. The details of the CART cell manufacturing process have been described in previous studies (16–19). Bridging treatment (platinum and/or anthracycline combinations with BTKi, ICPi, or BCL2i) was permitted prior to CD20CART infusion in cases of bulky disease (**Supplementary Figure S1**). Lymphodepleting chemotherapy was fludarabine (30 mg/m2, d-5 to d-3) monotherapy or in combination with cyclophosphamide (CTX, 300 mg/m2, d-5 to d-3). And if patients who have previously used fludarabine would receive lymphodepletion chemotherapy composed of bendamustine (90 mg/m2 d-5, -4). The infusion was performed on day 0. The infusion dose was administered at target number 2 ×10^6^cells/kg, minimum accepted dosage of CAR T cells (1 × 10^5^/kg) and maximum accepted dosage of CAR T cells (10×10^6^/kg).

Transduction efficiency and cell viability were examined at the time of CD20-SD-CART cell infusion. Transduction efficiency was defined as the ratio of CAR-T to CD3+ T cells, determined by flow cytometry (FCM). When the harvest of CD20 CAR T-cells was less than 1×10^5^/kg, we defined it as CAR manufacturing failure. A multicolor flow cytometer (FACSCalibur, BD, USA) was employed to detect CART cells on day 3 (d3), day 7, day 14, day 21, and day 28 post-transfusion. After that, monitoring continued monthly until the assay reached a lower quantitation limit. Enhanced CT/MRI for patient evaluation was performed once per month for the first six months. The first PET/CT was performed in the third month and then every 3 months until disease progression.

Non-CART therapy consisted of polatuzumab (pola-based) and non-polatuzumab (non-pola-based) therapies. Polatuzumab was given a dose of 1.6–1.8mg/kg intravenously every 3–4 weeks. The non-polatuzumab therapy in our study included lenalidomide, immune checkpoint inhibitors (ICPi), B cell lymphoma/leukemia-2 inhibitors (BLC2i), Bruton tyrosine kinase inhibitors (BTKi), and chemotherapy (anthracycline and/or platinum-based). The treatment regimen involved one cycle every 28 days, with enhanced CT or MRI assessment conducted before the next treatment cycle. Additionally, PET/CT monitoring was performed every two cycles.

### Outcomes

The primary outcome was overall survival (OS). Secondary outcomes included the best overall response rate (best ORR), progression-free survival (PFS), and the incidence of treatment-related adverse reactions. Response assessment was conducted according to the Lugano 2014 criteria, which categorized responses as complete remission (CR), partial remission (PR), stable disease (SD), or progression disease (PD) (20). The best ORR was defined as the percentage of patients achieving a response, including the complete remission rate (CRR) and partial remission rate (PRR). PFS in the CD20-SD-CART group was defined as the time from infusion of CD20CART cells to disease progression, last follow-up, or death. PFS in the non-CART group was defined as the time from initiating a new line of treatment after CD19 CART therapy to disease progression, last follow-up, or death. OS was defined as the time from the date of CD20CART cell infusion for the cellular group or the initiation of a new line of treatment for non-CART after CD19 CART therapy to the date of death or last follow-up. Treatment-related adverse reactions, such as cytokine release syndrome (CRS) and immune effector cell-associated neurotoxic syndrome (ICANS), were graded and managed according to the American Society of Transplant and Cellular Therapy grading criteria (21).

### Data collection

Medical records at the time of progression following CD19 CART therapy were screened. The following data for each patient were extracted: age, sex, medical histology, number of extranodal site lesions, bulky disease (bulky disease was defined as maximal diameter ≥7.5cm measured in either the transverse or coronal plane on computed tomography), Eastern Cooperative Oncology Group performance status (ECOG PS) (22), disease stage(Ann Arbor stage), International Prognostic Index (IPI) score (23), lactate dehydrogenase level (LDH), TP53 genes mutation by targeted next-generation sequencing, therapies pre-CD19 CART, response/nonresponse to CD19 CART, anti-cancer treatment therapy following CD19 CART progression, and clinical response. We used a combination of telephone and outpatient visits for follow-up observations and followed up to August 31, 2022.

### Statistical analysis

Analyses were performed by using SAS 9.4 (SAS Institute Inc, Cary, NC). The continuous data conforming to the normal distribution were expressed as means ± standard deviation and compared by independent sample t-test. When analyzing continuous variables that did not conform to a normal distribution, we described them using medians and IQR, and assessed the difference using the rank sum test. Categorical variables were reported in numbers and percentages and analyzed using the chi-square (χ^2^) or Fisher’s exact test. Logistic regression was used for multivariable analyses of the response. Estimates of survival were calculated according to the Kaplan-Meier method. Factors associated with PFS and OS were analyzed using Cox proportional hazards regression models. Factors entered the multivariable model according to clinical significance and the p-value of the univariate analysis (p<0.1). A two-sided P ≤ 0.05 was considered statistically significant.

## Results

### Patients and clinical characteristics

**Figure 1:**
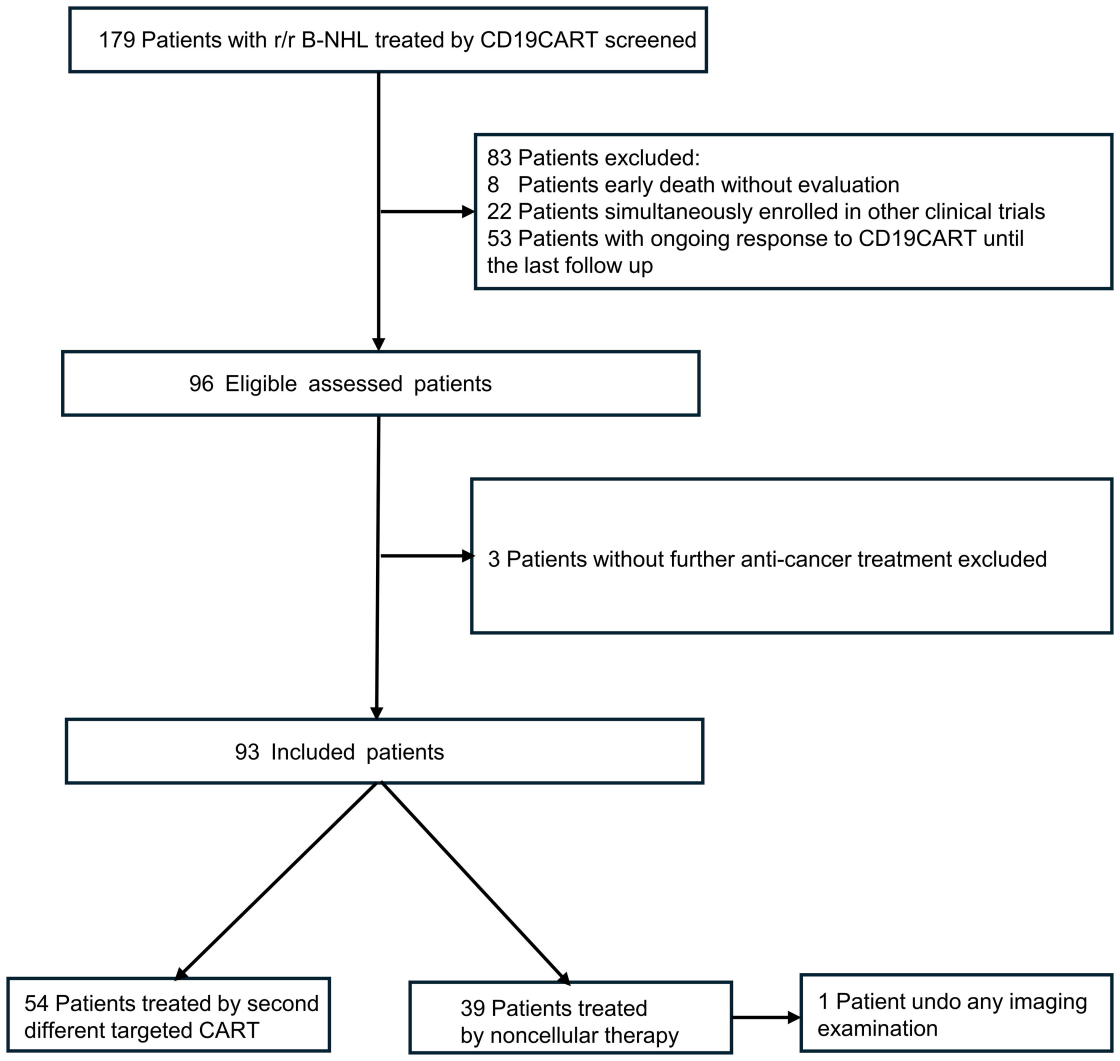
Flow diagram of patient enrollment.

A total of 93 patients who had previously undergone CD19 CART treatment were included in this study after careful screening from a pool of 179 patients (**Figure 1**). The median age of the included patients was 51 years (interquartile range [IQR], 42–64), and 52 (55.9%) were male. Among the 93 patients, 40 experienced relapsing after CD19 CART treatment (CR, n=13; PR, n=27), while 53 were refractory. After infusion, the median time to CD19 CART treatment failure was 2.3 months (IQR, 1.35–3.06). At progression or relapse following CD19CAR T-cell therapy, rebiopsies and CD19 evaluation by IHC/FCM were available in 49 patients (52.7%). 14 (28.6%) were found to be CD19-negative, while 35 (71.4%) remained CD19-positive. The median time of CD19CART treatment failure occurrence was 2.27 months (IQR, 1.25–3.31) in the CD19-negative group and 2.17 months (IQR, 1.48–2.87) in the CD19-positive group (p=0.56). Of the 93 patients, 54(relapsed n=23, refractory n=31) received CD20CART immunotherapy, while 39 (relapsed n=17, refractory n=22) received non-CART therapy including pola-based(n=15), targeted drugs (n=14: BCL2 inhibitors n=5, lenalidomide n=3, ICPi n=3, and BTKi n=3),and chemotherapy alone(n=10). The baseline characteristics of the patients are presented in **Table 1**. The two groups had no significant differences in baseline characteristics (all p>0.05).

**Table 1:** Baseline characteristics.

Variable, n (%)	CD20-SD-CART (n=54)	Non-CART (n=39)	p
**Age, years, Median, (IQR)**	50 (41–60)	55 (43–64)	0.27
**Sex, male**	28 (51.9)	24 (61.5)	0.47
ECOG PS			>0.99
** 0**	13 (24.1)	9 (23.1)	
** 1**	17 (31.5)	13 (33.3)	
** 2**	12 (22.2)	9 (23.1)	
** 3**	8 (14.8)	6 (15.4)	
** 4**	4 (7.4)	2 (5.1)	
**Disease stage (≥3)**	49 (90.7)	34 (87.1)	0.84
**IPI (≥3)**	29 (53.7)	20 (51.3)	0.98
LDH(>ULN) *	38 (82.6)	35 (89.7)	0.53
**No. of extranodal lesions (≥2) ^&^**	21 (39.6)	16 (41.0)	>0.99
**Bulky disease (≥7.5 cm) ^#^**	16 (31.4)	19 (48.7)	0.84
**BM involvement (yes)**	11 (20.4)	4 (10.3)	0.31
**CNS involvement (yes)**	7 (13.0)	2 (5.1)	0.37
Disease type			
** DLBCL NOS**	41 (75.9)	31 (79.5)	0.88
** HGBL**	7 (13)	3 (7.7)	0.64
** tFL**	1 (1.9)	3 (7.7)	0.39
** BL**	4 (7.4)	3 (7.7)	>0.99
COO^$^			0.19
** GCB**	27 (54.0)	13 (37.1)	
** Non-GCB**	23 (46.0)	22 (62.9)	
**Double/triple hit^**	6 (13.3)	3 (11.5)	>0.99
**TP53 gene mutation^@^**	24 (48.0)	13 (39.4)	0.58
**Prior lines (≥3)**	27 (50.0)	18 (46.2)	0.88
**No. of prior lines, Median, (IQR)**	8 (7–11)	9 (6–12)	0.41
**Prior SCT**	7 (13.0)	3 (7.7)	0.64
**Prior radiotherapy**	15 (27.8)	5 (12.8)	0.14
Type of CD19 CART treatment failure			>0.99
** Relapsed**	23 (42.6)	17 (43.6)	
** Refractory**	31 (57.4)	22 (56.4)	
**Median time of CD19 CART treatment failure occurred, Median, (IQR)**	2.3 (1.5–3.0)	2.0 (1.0–3.8)	0.7
**Time from CD19 CART infusion to failure occurrence (≤90d)**	40 (74.1)	26 (66.7)	0.59
CD19 status ^aΔ^			0.4
** CD19+**	26 (76.5)	9 (60.0)	
** CD19-**	8 (23.5)	6 (40.0)	

CART, chimeric antigen receptor T cell; ECOG PS, Eastern Cooperative Oncology Group performance status; IPI, international prognostic index; LDH, lactate dehydrogenase; BM, bone marrow, CNS, central nervous system, DLBCL NOS, diffuse large B-cell lymphoma not otherwise specified; GCB, germinal center B cell; HGBL, high-grade B-cell lymphoma; PMBCL, primary mediastinal B-cell lymphoma; BL, Burkitt lymphoma; MCL, mantle cell lymphoma; TFL, transformed follicular lymphoma; FL, follicular lymphoma; SCT, stem cell transplantation; BT, bridging treatment; CRS, cytokine release syndrome; ICANS, immune effector cell-associated neurotoxicity syndrome; ULN, upper limit of normal; PD, progressive disease; IHC/FCM, immunohistochemistry/flow cytometry.

Values are presented as numbers (%).

a CD19 expression at progressive disease after CD19 CART by immunohistochemistry/flow cytometry.

* Percentages do not include 8 patients in whom corresponding characteristics were unknown.

^&^ Percentages do not include 1 patient in whom the number of extranodal lesions was unknown.

Percentages do not include 3 patients in whom bulky disease was unknown.

^$^ Percentages do not include 8 patients in whom COO was not available/unknown.

^Percentages do not include 22 patients in whom DH/TH was unavailable.

Percentages do not include 10 patients in whom the TP53 gene was not unknown.

^Δ^ Percentages do not include 48 patients in whom CD19 status was unavailable.

### Best ORR, PFS, and OS of the entire cohort

**Figure 2:**
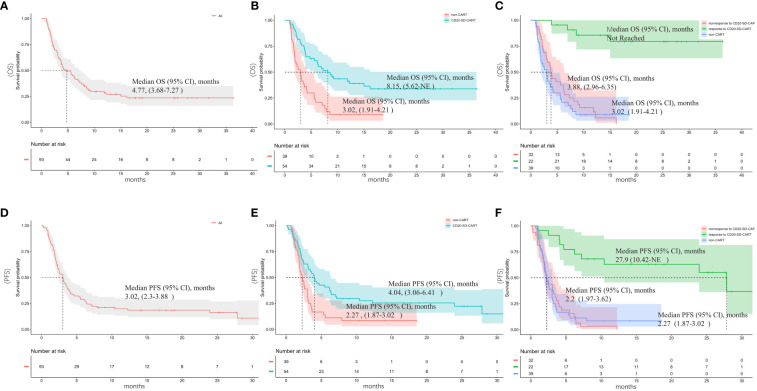
Outcomes after subsequent anti-cancer salvage therapy. **(A)** Overall survival (OS) and **(D)** progression-free survival (PFS) of the entire cohort; **(B)** Overall survival (OS) and **(E)** progression-free survival (PFS) of the CD20-SD-CART and non-CART group; **(C)** OS and **(F)** PFS of patients who responded to CD20-SD-CART, patients who did not respond to CD20-SD-CART and non-CART group.

Among the 93 patients enrolled in the study, one patient declined to undergo an imaging examination and was not rated. The best ORR(PRR+CRR) of the primary cohort was 32/92 (34.8%) and CRR was 18/92 (20%). Of patients who had no response to CD20CART(n=32), 19 (59.3%) ones were refractory to CD19CART. A median follow-up period of 18.54 months (95% confidence interval [CI], 14.2–25.8) was conducted for the patients included in this study. The overall median PFS and median OS were 3.02 months (95%CI, 2.3–3.88) and 4.77 months (95%CI, 3.68–7.27), respectively (**Figures 2A, D**). At the cutoff date, 17 (53.1%) patients had an ongoing response for the entire cohort, and 26 (81.3%) patients were still alive.

### Survival advantage of CD20-SD-CART therapy compared with non-CART

Comparisons were made between the CD20-SD-CART and non-CART groups in ORR, PFS, and OS. There was no significant difference observed in the best ORR between the CD20-SD-CART group (22/54, 40.7%) and the non-CART group (10/38, 26.3%) (p=0.15). However, the CRR in the CD20-SD-CART group (15/54, 27.8%) was significantly higher than that in the non-CART group (3/38, 7.9%) (p=0.03). Among patients treated with CD20-SD-CART, the median PFS was 4.04 months (95% CI, 3.06–6.41), and the median OS was 8.15 months (95% CI, 5.62-NE). In contrast, for patients receiving non-CART treatment, the median PFS was 2.27 months (95% CI, 1.87–3.02), and the median OS was 3.02 months (95% CI, 1.91–4.21) (**Figures 2B, E**). Notably, patients in the CD20-SD-CART group demonstrated significantly longer PFS (HR, 0.52; 95% CI, 0.32–0.86; p=0.003) and OS (HR, 0.38; 95% CI, 0.22–0.66; p<0.0001) compared to patients in the non-CART group. The Cox proportional hazard model was used to reduce confounding factors. Multivariate regression analysis revealed that the CD20-SD-CART has independently associated with better PFS (HR 0.46, 95% CI 0.27–0.8, p=0.005) and OS(HR 0.28, 95% CI 0.16–0.51, p<0.0001) (factors enter the cox model are shown in **Table 2**).

**Table 2:** Univariate and multivariate analyses of factors associated with survival following progression post-CD19 CART.

Variables	Univariable analysis				Multivariable analysis			
	OS		PFS		OS		PFS	
	HR (95% CI)	p	HR (95% CI)	p	HR (95% CI)	p	HR (95% CI)	p
**Age (>60)**	1.01 (0.59–1.74)	0.97	1.30 (0.78–2.14)	0.31	0.68 (0.37–1.27)	0.23	0.92 (0.51–1.65)	0.78
**sex (female)**	1.47 (0.91–2.38)	0.12	1.66 (1.05–2.61)	0.03				
**ECOG PS (≥3)**	3.49 (2.01–6.06)	<0.0001	3.49 (2.01–6.06)	<0.0001	4.11 (2.10–8.06)	<0.0001	3.78 (1.97–7.25)	<0.0001
**Disease Stage (≥3)**	1.04 (0.48–2.28)	0.9	1.00 (0.50–2.01)	0.99				
**IPI (≥3)**	1.70 (1.04–2.78)	0.03	1.76 (1.11–2.79)	0.02	1.1 (0.58–2.02)	0.81	1.17 (0.66–2.08)	0.6
LDH*	1.37 (0.65–2.89)	0.4	1.21 (0.62–2.37)	0.58				
**No. of Extranodal lesions (≥2)**	1.70 (1.05–2.76)	0.03	1.40 (0.88–2.23)	0.15				
**Bulky disease (≥7.5)**	1.78 (1.08–2.91)	0.02	1.36 (0.85–2.18)	0.2				
**BM involvement (yes)**	1.00 (0.53–1.92)	0.99	0.91 (0.49–1.7)	0.77				
**CNS involvement (yes)**	0.96 (0.41–2.21)	0.92	0.97 (0.45–2.13)	0.95				
**COO (GCB)**	0.56 (0.33–0.95)	0.03	0.80 (0.49–1.30)	0.36	0.73 (0.42–1.27)	0.27	0.95 (0.57–1.58)	0.85
**Double/triple-hit (yes)**	1.54 (0.72–3.27)	0.3	1.82 (0.86–3.86)	0.12				
**Prior lines (≥3)**	0.69 (0.42–1.11)	0.1	0.68 (0.43–1.07)	0.1				
**Prior SCT (yes)**	0.35 (0.14–0.89)	0.03	0.46 (0.21–1.01)	0.05	0.4 (0.14–1.20)	0.1	0.54 (0.22–1.3)	0.17
**Prior irradiation (yes)**	0.95 (0.54–1.67)	0.86	0.78 (0.45–1.35)	0.37				
**Response to CD19 (refractory vs. relapsed)**	1.37 (0.83–2.26)	0.2	0.99 (0.63–1.57)	0.98				
CD19 status^a^ (positive)	0.55 (0.28–1.07)	0.08	0.51 (0.26–0.99)	0.05				
**treatment type (CD20-SD-CART)**	0.36 (0.22–0.59)	<0.0001	0.5 (0.31–0.80)	0.004	0.28 (0.16–0.51)	<0.0001	0.46 (0.27–0.8)	0.005
					0.28 (0.16–0.51)	<0.0001		

OS, overall survival; PFS, progression-free survival; HR, hazard ratio; COO, cell of origin; GCB, germinal center B cell; SCT, stem cell transplantation; BM, bone marrow; CNS, central nervous system. ECOG PS, Eastern Cooperative Oncology Group performance status; IPI, international prognostic index.

* LDH levels at progression after CD19-CART.

a CD19 expression at progressive disease after CD19 CART by immunohistochemistry/flow cytometry.

**Figure 3:**
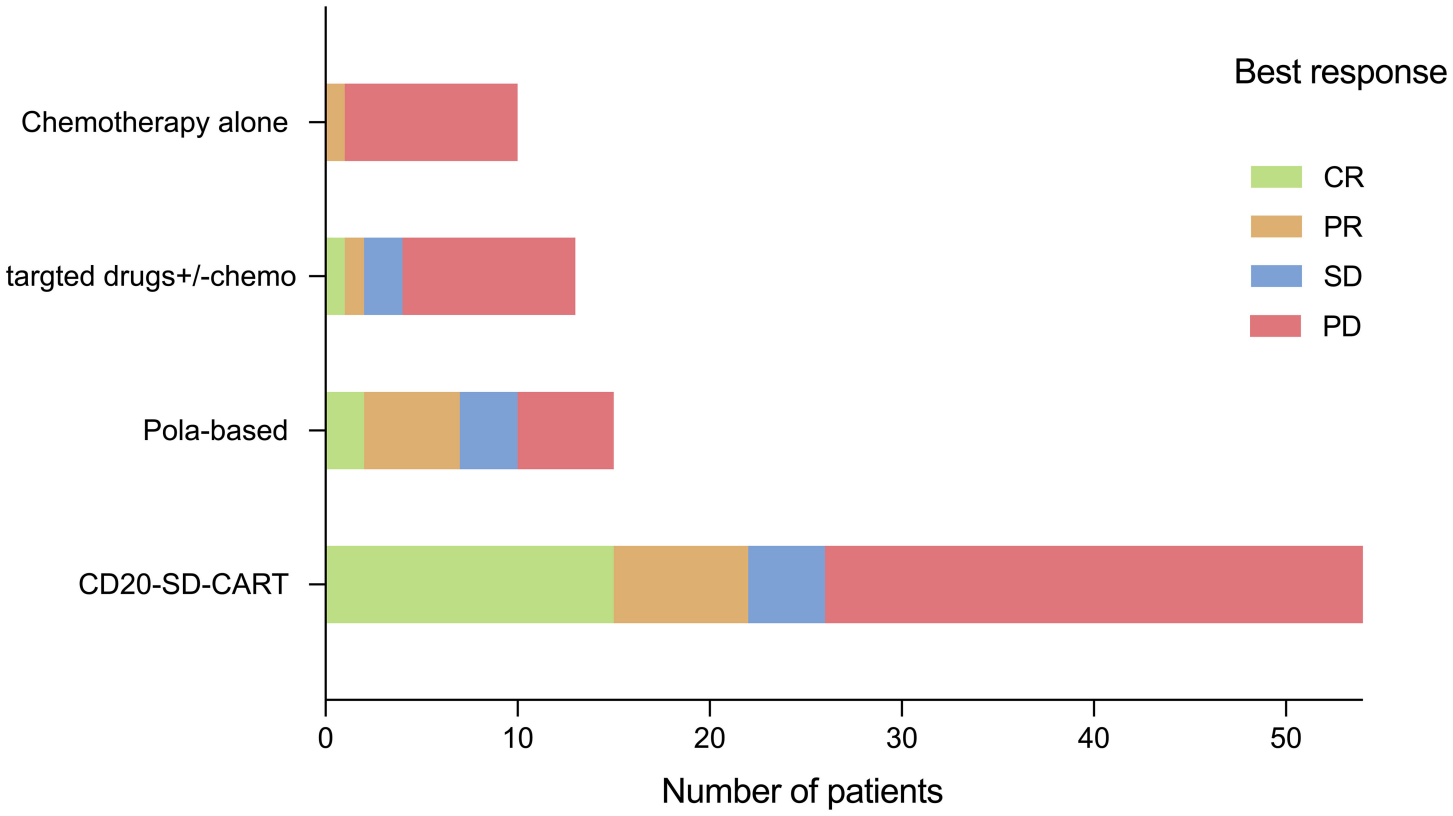
Outcomes of the CD20-SD-CART therapy and non-CART therapy.

Among the 39 patients who received non-CART treatment, 15 patients (38.5%) received pola-based therapy. In the pola-based group, the best ORR was 7/15 (46.7%) with a CRR of 2/15(13.3%) (**Figure 3**). When comparing the pola-based group to the CD20-SD-CART group, no statistical differences were observed in ORR (46.7% vs. 40.7%, P=0.77), CRR (13.3% vs. 27.8%, P=0.33), PFS(3.62months vs. 4.04 months p=0.69), and OS(5.59 months vs. 8.15 months p=0.28). However, a trend for CD20-SD-CART to be more effective was observed (**Supplementary Figure S2**). Additionally, among patients with the ongoing response (n=17) at the cutoff day, 12(70.6%) were in the CD20-SD-CART group, whereas 3(17.6%) were in pola-based group. Among the survivors (n=26), 18(69.2%) were in the CD20-SDCART group, while 5 were in pola-based group.

We also found that in the CD20-SD-CART group, the PFS for the 22 responders was 27.9 months (95% CI, 10.42-NE), while that for the 32 non-responders was only 2.2 months (95% CI, 1.97–3.62) (HR, 0.14, 95% CI 0.06–0.3, p<0.0001). The median OS for the responders to CD20-SD-CART was not reached, while that for the non-responders was only 3.88 months (95% CI, 2.96–6.35) (HR 0.08, 95% CI 0.03–0.24, p<0.0001). No statistically significant difference was found in the median PFS (HR 1.05, 95% CI 0.64–1.71, p=0.86) and OS (HR 0.74, 95% CI 0.45–1.23, p=0.24) between patients who did not respond to CD20-SD-CART therapy and those in the non-CART group. However, for patients who responded to CD20-SD-CART, the median PFS (HR 0.14, 95% CI 0.06–0.31, p<0.0001) and OS (HR 0.06, 95% CI 0.02–0.18, p<0.0001) were significantly longer compared to the non-CART group (**Figures 2C, F**).

### Features of CD20-SD-CART therapy

Among the 54 patients treated with CD20-SD-CART therapy, 37 (68.5%) received bridging treatment with a CRR of 1/37 before starting lymphodepletion chemotherapy. Moreover, 49 patients (90.7%) received lymphodepletion chemotherapy (Flu-based n=32, Ben-based n=17). The median infusion dose was 1.21 ×10^6^cells/kg (IQR, 0.4–2.1). The infusion dose for 37 patients (68.5%) fell below the target level. After CD20-SD-CART infusion, CART cells were detectable in the peripheral blood by FCM from most patients (45/54), indicating a high *in vivo* expansion rate. The expansion peaked from days 12 to 15 after infusion, and the median expansion peak of CD20-SD-CART cells reached 7.76×10^6^cells/L (IQR 2.56–29.65). Despite receiving a comparable CD20-SD-CART infusion dose to CD19 CART (1.21 ×10^6^cells/kg vs.1.28×10^6^cells/kg, p=0.65), we observed lower CART cell peak expansion after CD20-SD-CART than that after CD19 CART (7.76×10^6^cells/L vs. 52.6×10^6^cells/L, p<0.0001) (**Supplementary Figures S1A, B**).

Concerning the safety of CD20-SD-CART, we found that 5 patients (9.2%) experienced severe CRS (≥grade 3), and 4 patients (7.4%) had severe ICANS (≥grade 3) (**Supplementary Figures S1C, D**). Dexamethasone was administered to treat CRS and/or ICANS in 13 patients (24%) after CD20-SD-CART. The patients who developed severe ICANS and CRS completely recovered, and no treatment-related deaths occurred. We noted comparable proportions of patients with sCRS (9.2% vs. 10.9%, p>0.999) and proportions of patients who developed sICANS (7.4% vs. 8.7%, p>0.999) between the CD20-SD-CART and CD19 CART.

Serum cytokine markers of systemic inflammation such as interleukin 6(IL-6), tumor necrosis factor-α (TNFα), and ferritin, exhibited elevation during CAR T-cell treatment. The changes in cytokines (IL6, TNFα, IL10, and ferritin) are shown in **Supplementary Figure S4**. Toxicities assessed by CTCAE 5.0 grading were depicted in **Supplementary Figure S4**. Overall, CD20-SD-CART regimens did not escalate toxicity, and no Grade 5 events were reported.

### Risk factors associated with the outcome of SD-CART

Using a univariable logistic regression model to assess factors related to the response of CD20-SD-CART, the findings showed that a higher infusion dose (OR 1.74, 95% CI, 1.1 - 2.86, p=0.03) was associated with a higher ORR, while IPI score ≥3 (OR 0.3, 95% CI, 0.10 - 0.93, p=0.04) and bridging treatment before CD20-SD-CART (OR 0.23, 95% CI, 0.07 - 0.78, p=0.02) were associated with a lower ORR. However, no correlation with response was detected when these factors were incorporated in a multifactor logistic regression (**Supplementary Table S1**). Further multivariable analyses of the PFS and OS in CD20-SD-CART group illustrated that shorter PFS was associated with ECOG PS ≥3(HR, 3.12, 95% CI, 1.23–7.94, p=0.02), while longer PFS was associated with increased infusion dose (HR, 0.68, 95% CI, 0.5–0.92, p=0.01). Additionally, the OS was associated with ECOG ≥3 (HR, 4.13, 95% CI, 1.64–10.5, p=0.003) and refractory to CD19 CART (HR, 2.19, 95% CI, 1.01–4.76, p=0.0047) (**Table 3**).

**Table 3:** Univariate and multivariate analyses of factors associated with PFS and OS of CD20-SD-CART.

Variables	Univariable analysis				Multivariable analysis			
	OS		PFS		OS		PFS	
	HR (95% CI)	p	HR (95% CI)	p	HR (95% CI)	p	HR (95% CI)	p
**Age (>60)**	1 (0.45–2.20)	0.99	1.67 (0.83–3.36)	0.15				
**Sex (male)**	0.66 (0.33–1.31)	0.2	0.67 (0.36–1.24)	0.2				
**Stage (≥3)**	1.89 (0.45–7.93)	0.38	1.28 (0.45–3.63)	0.64				
**IPI score (≥3)**	2.22 (1.09–4.52)	0.03	2.09 (1.11–3.95)	0.02	1.56 (0.66–3.67)	0.31	1.96 (0.91–4.23)	0.09
**ECOG PS (≥3)**	5.52 (2.54–12)	<0.0001	5.02 (2.3–10.9)	<0.0001	4.13 (1.64–10.5)	0.003	3.12 (1.23–7.94)	0.02
**LDH (>ULN)**	0.85 (0.34–2.11)	0.73	0.74 (0.33–1.63)	0.45				
**No. of Extra nodals (≥2)**	2.35 (1.18–4.66)	0.02	1.89 (1.00–3.54)	0.05				
**Bulky disease (≥7.5)**	1.78 (0.87–3.65)	0.12	1.2 (0.61 -2.34)	0.61				
**Double/triple hit**	2.02 (0.77–5.31)	0.15	1.93 (0.74–5.01)	0.18				
**TP53 gene mutation**	1.95 (0.96–3.97)	0.07	1.42 (0.75–2.71)	0.28				
**CD19 status post-CD19 CART (positivity)**	0.72 (0.30–1.73)	0.47	0.66 (0.28–1.56)	0.35				
**Response to CD19 (refractory)**	1.72 (0.84–3.53)	0.14	1.02 (0.55–1.89)	0.96	2.19 (1.01–4.76)	0.047	1.48 (0.77–2.85)	0.24
**BT preCD20-SD-CART^#^**	2.12 (0.92–4.91)	0.08			1.25 (0.50–3.14)	0.63	0.85 (0.40–1.80)	0.67
**CD20-SD-CART Infusion dose**	0.77 (0.58–1.03)	0.08			0.81 (0.59–1.12)	0.2	0.68 (0.50–0.92)	0.01
CART interval*	0.83 (0.7–0.99)	0.04						
**Exposure to cortisone in CD20-SD-CART (yes)**	1.1 (0.5–2.45)	0.81						

OS, overall survival; PFS, progressive free survival; HR, hazard ratio; COO, cell of origin; GCB, germinal center B cell; SCT, stem cell transplantation; ECOG PS, Eastern Cooperative Oncology Group performance status; IPI, international prognostic index.

*Interval between CD19 CART infusion and CD20-SD-CART Infusion.

^#^The bridging therapies employed novel agents, including BCL2 inhibitors (n=27), BTKi (n=18), lenalidomide (n=7), and XPO1 inhibitors (n=3), individual or in combination with chemotherapy.

## Discussion

The results showed that patients who received CD20-SD-CART infusion had longer PFS and OS compared to those who received non-CART therapy, suggesting that CD20-SD-CART may serve as a promising salvage therapy for aBCL patients after failure of CD19 CART treatment.

In this study, the ORR (22/54) and CRR (15/54) were lower than in another clinical trial of patients with CD19 CART treatment failure treated by CD22 CART (ORR, 6/7; CRR, 4/7), it may be due to small sample size(n=7) (24). However, both studies have demonstrated that employing an alternative targeted CART therapy as a secondary treatment for cancer, in cases where CD19 CART has proven ineffective, is a viable and efficacious option. This positive outcome may be attributed to the underlying mechanisms of CD19 CART treatment failure:1) inadequate persistence of CART cells in the body, leading to resistance to CD19 CART cell therapy (25, 26), and 2) mutations, loss, or downregulation of the CD19 antigen, which impairs the ability of CART cells to recognize and eliminate cancer cells due to the absence of target antigens (27, 28). In our study, 49 patients were rebiopsied after CD19 CART failure, and CD19 status was reassessed. 35 patients had CD19-positive recurrence, and 14 had CD19-negative recurrence, which was like the results of Spiegel et al. and Tomas et al. (6, 7). Whether the recurrence was CD19 negative, applying a second different targeted CART enhanced CART-mediated recognition and clearance due to plenty of targeting antigens on the surface of B-cell malignancies (27, 28). In our study, it is interesting to note that among the 32 patients unresponsive to CD20-SD-CART, 19 (59.3%) showed refractory to CD19 CART, while 13 (40.6%) experienced a relapse after CD19 CART, potentially connected to immune escape. Further research is needed to understand the mechanisms involved.

Compared to non-CART therapy, CD20-SD-CART had a survival advantage as a salvage therapy after CD19 CART treatment failure. In the present study, the CD20-SD-CART group had a longer PFS (p=0.0032) and OS (p<0.0001). After Cox multivariate regression analysis, the association remained evident (**Table 2**). These results suggest CD20-SD-CART may provide a potential treatment strategy for some patients following CD19CART failure. Regrettably, in our data, bispecific antibodies (such as Mosunetuzumab and Glofitamab which are not yet available in China) were not utilized in the non-CART therapy; this absence may introduce some bias into the results. However, Blasi et al. analysis on bispecific antibody administration in CD19CART treatment failure context revealed that both the ORR and CRR were 14.3% (n=11), which was significantly lower than the 44% of polatuzumab reported by Gouni et al. (n=55). Additionally, Tomas et al. reported that 135 patients received subsequent anti-cancer including polatuzumab-based (n = 29), chemotherapy approaches (n = 17; anthracycline or platinum-based), lenalidomide-based (n = 15), involved site radiation therapy (ISRT) monotherapy (n = 15), and Bruton’s tyrosine kinase inhibitor-based (BTKi; n = 14), checkpoint inhibitors (n=10). It has been demonstrated that pola-based therapy was the better treatment strategy among non-CART therapies in the CD19 CART treatment failure (the ORR:48%, CRR:34%) (7, 10). Similarly, in our experience, we also observed that pola-based therapy yielded the higher ORR of 46.7% among the non-CART therapies (**Figure 3**). Nonetheless, our findings, along with those of Alarcon Tomas et al., showed that pola-based treatment exhibits a brief response duration among patients experiencing relapse or progression following CD19CAR T therapy. The response did not translate into prolonged 1-year post-treatment-OS, which may be another reason why non-CART therapy had no survival advantage compared to CD20-SD-CART. Although there was no statistical difference in objective response rate (P=0.77), complete response rate (P=0.33), PFS (p=0.69), and OS (p=0.28) between CD20-SD-CART therapy and pola-based therapy, which may be an association with smaller sample size, a trend for CD20-SD-CART to be more effective than other therapies was observed.

In the present study, it was also observed that patients who exhibited unresponsiveness to CD20-SD-CART demonstrated comparable survival rates to those in the non-CART treatment cohort. However, those who responded to CD20-SD-CART had better PFS and OS than those who did not respond to CD20-SD-CART or who were treated by non-CART therapy (**Figures 2C, D**). These findings suggest that CD20-SD-CART therapy may play a role in sustaining an ongoing response. In instances where CD19 CART therapy has proven ineffective, CD20-SD-CART may provide a way to regain sustained remission. Additionally, regarding safety, CD20-SD-CART administration did not seem to increase the incidence of CRS or severe CRS, nor were ICANS and severe ICANS (**Supplementary Figures S3C, D**). There is no treatment related to death.

In addition, further analysis of factors associated with outcomes of the CD20-SD-CART showed that patients with worse ECOG performance status (ECOG≥3), with refractory to CD19 CART, and with fewer infusion doses may not benefit from CD20-SD-CART immunotherapy.

Our study has several limitations beyond its retrospective nature. Firstly, this study was a single-center study and has a limited number of patients, which may limit the generalizability of the findings. Secondly, the patients included in the study underwent various treatment strategies, which introduces the possibility of treatment heterogeneity. This heterogeneity may contribute to some degree of bias in the results during statistical analysis despite our efforts to mitigate this through appropriate statistical methods.

Thirdly, in the study, only 49 out of 93 patients (52.7%) underwent re-biopsy at progression post-CD19 CART, potentially introducing information bias. Enhancing patient compliance and increasing the re-biopsy rate are imperative measures to address this concern. Fourthly, Selection of treatment regimens was guided by factors such as CD20 expression and ECOG performance status, which could act as latent confounders and potentially introduce selection bias between treatments. However, we have extensively characterized the differences among the groups and restricted comparisons to only the most common treatments while adjusting for key known confounders to mitigate this bias. Finally, the use of anti-cancer non-CART therapy without bispecific antibody drugs such as Mosunetuzumab and Glofitamab involvement was a potential confounder. Further multicenter, prospective, larger sample size and randomized studies are encouraged to confirm the benefits of CD20-SD-CART on survival after CD19 CART treatment failure in the long-term follow-up.

## Conclusions

Compared to non-CART therapy, CD20-SD-CART demonstrates advantages in terms of survival outcomes and shows promising long-term efficacy. Our preliminary findings indicate that CD20-SD-CART could serve as a potential alternative for patients with relapsed or refractory aggressive B-cell lymphoma after CD19 CART therapy.

## Data availability statement

The original contributions presented in the study are included in the article/**Supplementary Material**. Further inquiries can be directed to the corresponding authors.

## Ethics statement

The studies involving humans were approved by Beijing Gobroad Boren Hospital. The studies were conducted in accordance with the local legislation and institutional requirements. The participants provided their written informed consent to participate in this study.

## Author contributions

FX: Conceptualization, Methodology, Project administration, Resources, Writing – original draft, Writing – review & editing. FY: Data curation, Investigation, Project administration, Writing – original draft, Writing – review & editing. PZ: Formal analysis, Methodology, Writing – original draft, Writing – review & editing. RL: Data curation, Formal analysis, Writing – original draft, Writing – review & editing. SF: Investigation, Supervision, Writing – original draft, Writing – review & editing. YG: Methodology, Project administration, Writing – original draft, Writing – review & editing. HS: Formal analysis, Methodology, Writing – original draft, Writing – review & editing. LM: Formal analysis, Methodology, Writing – original draft, Writing – review & editing. BD: Formal analysis, Writing – original draft, Writing – review & editing. TX: Formal analysis, Resources, Writing – original draft, Writing – review & editing. JZ: Writing – review & editing, Formal analysis, Visualization, Writing – original draft. QZ: Data curation, Formal analysis, Writing – original draft, Writing – review & editing. XK: Data curation, Formal analysis, Writing – original draft, Writing – review & editing. KH: Data curation, Formal analysis, Resources, Writing – original draft, Writing – review & editing.

## Supplementary material

Supplementary Figure 1 Treatment schema for aggressive B cell lymphoma with CD19CART failure.

Supplementary Figure 2 OS **(A)** and PFS **(B)** of CD20-SD-CART and pola-based group.

Supplementary Figure 3 Comparison between CD19 CART and CD20-SD-CART. **(A)** CART infusion (x10^6/kg) and **(B)** CART cell expansion (x10^6/L) comparison by flow cytometry. **(C)** Cytokine release syndrome grade (CRS), and **(D)** immune effector cell-associated neurotoxicity syndrome grade (ICANS) of CD19 CART and CD20-SD-CART. **(A, B)** Bold horizontal lines, median; box, IQR; vertical lines, quartiles ± 1.5×IQR. * p<0.0001.

Supplementary Figure 4 The changes in cytokines (IL-6, TNFα and ferritin). **(A)**, **(C)**, and **(E)** IL-6, TNFα, and ferritin changed after CD20-SD-CART infusion, respectively. **(B)**, **(D)**, and **(F)** IL-6, TNFα and ferritin peak level were 50.53pg/ml, 29.89pg/ml and 2090pg/ml, respectively. The median peak time was 7 days (IQR of 3 to 10), 7 days (range of 3–14), and 14 days (range of 7 to 14) after CART-cell transfusion, respectively. IL-6, interleukin 6, TNFα, tumor necrosis factor-α.

Supplementary Figure 5 Toxicities assessed by CTCAE grading were evaluated.

Supplementary Table 1 Univariate and multivariate analyses of factors associated with the response to CD20-SD-CART.
