# Supplementary material for: Salvage CD20-SD-CART therapy in aggressive B-cell lymphoma after CD19 CART treatment failure
Source: Front Oncol. 2024 Jun 25;14:1376490. doi: 10.3389/fonc.2024.1376490 (PMC11232503; doi:10.3389/fonc.2024.1376490)
Supplement: Supplementary Figure 1 — Treatment schema for aggressive B cell lymphoma with CD19CART failure. [file DataSheet_1.docx]

**Table S1.** Univariate and multivariate analyses of factors associated with the response to CD20-SD-CART

| Variables | Univariate analysis | | Multivariate analysis | |
| --- | --- | --- | --- | --- |
|  | OR (95% CI) | p | 95% CI | p |
| Age (>60) | 0.54 (0.14-2.06) | 0.37 |  |  |
| Sex (male) | 0.82 (0.28-2.46) | 0.73 |  |  |
| Stage (≧3) | 0.42 (0.06 - 2.76)) | 0.37 |  |  |
| IPI score (≧3) | 0.30 (0.10 - 0.93) | 0.04 | 0.03 (0.00 - 1.63) | 0.09 |
| ECOG PS (≧3) | - | 0.99 |  |  |
| LDH (>ULN) | 5.09 (0.57 - 45.58) | 0.15 |  |  |
| No. of Extra nodals (≧2) | 0.45 (0.14 - 1.47) | 0.19 |  |  |
| Bulky (≧7.5) | 0.40 (0.11 - 1.47) | 0.17 |  |  |
| Double/triple hit | 0.26 (0.03 - 2.43) | 0.24 |  |  |
| TP53 gene mutation | 0.58 (0.19 - 1.84) | 0.36 |  |  |
| CD19 status postCD19 CART (positive) | 3.71 (0.39 - 35.01) | 0.25 |  |  |
| Response to CD19 CART (refractory) | 0.82 (0.27-2.44) | 0.72 |  |  |
| BT preCD20-SD-CART^#^ | 0.23 (0.07 - 0.78) | 0.02 | 0.05 (0.00 - 2.28) | 0.12 |
| CD20-SD-CART Infusion dose | 1.74 (1.06 - 2.86) | 0.03 | 3.29 (0.69 - 15.70) | 0.14 |
| CART interval* | 1.13 (0.93 - 1.37) | 0.23 |  |  |
| Exposure to cortisone in CD20-SD-CART (yes) | 1.34 (0.38 - 4.71) | 0.65 |  |  |

OS, overall survival;PFS, progressive free survival; OR, odds ratio;DLBCL, diffuse large B-cell lymphoma;HGBCL, high-grade B-cell. lymphoma;PMBCL, primary mediastinal B-cell lymphoma; BL, Burkitt lymphoma; MCL, mantle cell lymphoma;TFL, transformed follicular lymphoma; FL, follicular lymphoma; COO,cell of origin; GCB, germinal center B cell; SCT,stem cell transplantation; CRS,cytokine release syndrome; ICANS, Immune effector cell-associated neurotoxicity syndrome; BM,bone marrow; CNS, central nerve system; ECOG PS,Eastern Cooperative Oncology Group performance status ; IPI, international prognostic index.

*Interval between CD19 CART infusion and CD20-SD-CART Infusion,

^#^The bridging therapies employed novel agents, including BCL2 inhibitors (n=27), BTKi (n=18), lenalidomide (n=7), and XPO1 inhibitors (n=3), individual or in combination with chemotherapy.

Figure S1 Treatment schema for aggressive B cell lymphoma with CD19CART failure. CD20-SD-CART cells, manufactured with cryopreserved autologous peripheral blood mononuclear cells (PBMC) stored when enrolled to CD19CART therapy, were prepared 6-7 days before infusion. Within this time, some patients were given lymphocytes depleting chemotherapy as described. Enhanced CT/MRI for patient evaluation was performed once per month for the first six months. The first PET/CT was performed in the third month and then every 3 months until disease progression. If bulky disease, we use of bridging chemotherapy to reduce the risk of severe adverse effects.





**Figure S2.** OS(A) and PFS (B) of CD20-SD-CART and pola-based group.

**Figure S3.** Comparison between CD19 CART and CD20-SD-CART. (A) CART infusion (x10^6/kg) and (B) CART cell expansion (x10^6/L) comparison by flow cytometry. (C) Cytokine release syndrome grade (CRS), and (D) immune effector cell-associated neurotoxicity syndrome grade (ICANS) of CD19 CART and CD20-SD-CART. (A-B) Bold horizontal lines, median; box, IQR; vertical lines, quartiles±1.5×IQR. * p<0.0001.

**Figure S4.** The changes in cytokines (IL-6, TNFα and ferritin). (A),(C),and(E) IL-6, TNFα and ferritin changed after CD20-SD-CART infusion, respectively.(B),(D),and (F) IL-6, TNFα and ferritin peak level were 50.53pg/ml, 29.89pg/ml and 2090pg/ml, respectively. The median peak time was 7 days (IQR of 3 to 10), 7 days (range of 3–14), and 14 days (range of 7 to 14) after CART-cell transfusion, respectively. IL-6, interleukin 6, TNFα, tumor necrosis factor-α.

A

B

**Figure S5.** Toxicities assessed by CTCAE grading were evaluated.
